# Young people's views on the potential use of telemedicine consultations for sexual health: results of a national survey

**DOI:** 10.1186/1471-2334-11-285

**Published:** 2011-10-25

**Authors:** Cameryn C Garrett, Jane Hocking, Marcus Y Chen, Christopher K Fairley, Maggie Kirkman

**Affiliations:** 1 Centre for Women's Health, Gender and Society, Melbourne School of Population Health, The University of Melbourne, Victoria 3010 Australia; 2 Melbourne Sexual Health Centre, Alfred Hospital, 580 Swanston Street, Carlton, Victoria 3053 Australia; 3 Melbourne School of Population Health, The University of Melbourne, Victoria 3010 Australia; 4 The Jean Hailes Research Unit, School of Public Health and Preventative Medicine, Monash University, Clayton, Victoria, 3168 Australia

## Abstract

**Background:**

Young people are disproportionately affected by sexually transmissible infections in Australia but face barriers to accessing sexual health services, including concerns over confidentiality and, for some, geographic remoteness. A possible innovation to increase access to services is the use of telemedicine.

**Methods:**

Young people's (aged 16-24) pre-use views on telephone and webcam consultations for sexual health were investigated through a widely-advertised national online survey in Australia. Descriptive statistics were used to describe the study sample and chi-square, Mann-Whitney U test, or t-tests were used to assess associations. Multinomial logistic regression was used to explore the association between the three-level outcome variable (first preference in person, telephone or webcam, and demographic and behavioural variables); odds ratios and 95%CI were calculated using in person as the reference category. Free text responses were analysed thematically.

**Results:**

A total of 662 people completed the questionnaire. Overall, 85% of the sample indicated they would be willing to have an in-person consultation with a doctor, 63% a telephone consultation, and 29% a webcam consultation. Men, respondents with same-sex partners, and respondents reporting three or more partners in the previous year were more willing to have a webcam consultation. Imagining they lived 20 minutes from a doctor, 83% of respondents reported that their first preference would be an in-person consultation with a doctor; if imagining they lived two hours from a doctor, 51% preferred a telephone consultation. The main objections to webcam consultations in the free text responses were privacy and security concerns relating to the possibility of the webcam consultation being recorded, saved, and potentially searchable and retrievable online.

**Conclusions:**

This study is the first we are aware of that seeks the views of young people on telemedicine and access to sexual health services. Although only 29% of respondents were willing to have a webcam consultation, such a service may benefit youth who may not otherwise access a sexual health service. The acceptability of webcam consultations may be increased if medical clinics provide clear and accessible privacy policies ensuring that consultations will not be recorded or saved.

## Background

Young people are disproportionately affected by sexually transmissible infections (STIs) [1]. Untreated STIs can have serious health consequences including infertility, ectopic pregnancy, and pelvic inflammatory disease in women [2]. As most STIs are asymptomatic, periodic screening for certain STIs, such as chlamydia, is recommended [2]; this requires adequate access to sexual health services.

Young people may face barriers to accessing sexual health services, including concerns over confidentiality and privacy, cost, limited transport, and too few medical providers [3-6]. Living in an isolated or remote region can also limit young people's options because there may be no available sexual health specialist and no choice of male or female doctor. Many young women in Australia prefer speaking to a doctor of the same sex [4,5,7]. Adolescents have reported concerns about being identified entering a clinic and that medical staff might disclose to others the reason for their visit [3,5]. In both rural and urban areas, concerns over the implications of sexual activity and, in particular, the stigma surrounding STIs have been reported to limit willingness to seek medical care for sexual health [6]. These findings highlight the importance of access to confidential services.

One possible means to increase access is the use of telemedicine. Telemedicine is defined as "the delivery of health services when there is geographic separation between health-care provider and patients, or between health-care professionals" [8]. Telemedicine itself falls into a broad category incorporating a range of technologies such as telephone, facsimile, and webcam consultations over the computer (also referred to as video consultations or videoconferencing) [9,10]. For the purpose of this paper, telemedicine refers to communication between patients and medical professionals. Webcam consultations in Australia have been used successfully in fields such as psychiatry, emergency care, and paediatrics [9,11].

Reviews of telemedicine between patients and medical professionals have cited numerous advantages for patients including increased access to services and providers, lessened travel and waiting time to see a doctor, and reduced cost [9,12,13]. Despite these advantages, concern has been raised about the quality of doctor-patient communication during telemedicine consultations, as well as about privacy and security [8,13].

Webcam or telephone consultations between health care providers and patients would enable young people to consult a doctor from their home computer or smart phone, obviating the need for a clinic visit and increasing their options around medical providers. After a consultation, a home STI testing kit could be posted to patients. These kits have been found to be reliable and acceptable STI screening tools [14,15].

We initiated a literature review in July 2009 to examine what was known about telephone and webcam consultations (video consultation or videoconferencing) for STI care between patients and health care professionals. A comprehensive search of the published peer-reviewed literature via Scopus, MEDLINE, Web of Science, PsycINFO, PubMed, and Academic Search Complete yielded no articles about using webcam consultations for STI care between patients and providers. Only one article was retrieved that dealt (indirectly) with the use of telephone consultations for STI care [16]. This research study intended to fill this void in the literature.

The aim of this study was to examine young adults' pre-use views on webcam and telephone consultations for sexual health in Australia.

## Methods

### Study Respondents

Young people aged 16-24, living in Australia, with Internet access were eligible to participate in the study.

### Instrument

An online questionnaire was deemed most appropriate to examine the relationship between health care and the Internet. In the absence of a standardised questionnaire about pre-use views on telemedicine, a study-specific questionnaire was devised; where appropriate, questions were adapted from other published questionnaires [17-19]. The national, cross-sectional SHOUT (Sexual Health Online Using Telemedicine) questionnaire had five sections: information about the respondents, their access to health care, discussing their sexual health with a doctor, IT information, and sexual behaviour. Respondents were asked their general views on webcam, telephone, and in-person consultations. Five-point Likert scales (very willing → very unwilling) were used to assess people's willingness to have a consultation by these different media. In addition to the fixed-answer questions, respondents could provide additional or explanatory comments in the free text response boxes. Next, respondents were asked to nominate their first preference for speaking to a doctor for an asymptomatic sexual health consultation if given the choice between an in-person, telephone, and webcam consultation. For this question, respondents were instructed to imagine two possible situations: living twenty minutes or two hours from a doctor. The questionnaire was accessible on the research study's website [20]. After piloting the questionnaire with urban and rural Australian young people, it was available to complete anonymously online from September 2009 to May 2010.

### Recruitment and Advertising

The survey used convenience sampling. Advertising was concurrent with the questionnaire's availability (9 months). A variety of advertising approaches was used: through universities, Australian organisations targeting young people, Facebook, and radio. A total of 105 diverse youth organisations across Australia were contacted about placing survey information on their website and/or newsletter; 11 (10%) (predominantly government-affiliated and rural organisations) agreed to advertise. Advertisements were placed on the University of Melbourne's online Student Portal Notice Board. Facebook groups targeting Australian youth were also contacted about posting information about the study on their Facebook page and a paid Facebook advertisement was placed online. Of the 77 Facebook groups contacted, 16 (21%) (mainly university groups or Facebook groups aimed at people living in rural areas) agreed to advertise. Contacting Facebook groups also resulted in advertisements in related blogs and newsletters. Of the 1855 people who clicked on the paid Facebook advertisement, 24 (1%) completed the questionnaire.

### Analysis

Descriptive statistics were used to describe the study sample. Variables based on Likert scales were collapsed into binary outcomes. Chi-square tests were used to assess associations of categorical variables, and t-tests or Mann-Whitney U tests were used to assess associations between binary and continuous variables. Multinomial logistic regression was used to explore the association between the three-level outcome variables (first preference in person, telephone or webcam, and demographic and behavioural variables); odds ratios and 95% CI were calculated using in-person consultation as the reference category. Free text responses were analysed thematically.

### Ethics

The study was approved by the University of Melbourne Human Research Ethics Committee (#931507).

## Results

### Sample Characteristics

A total of 662 people completed the questionnaire. Forty four percent of respondents (n = 289) wrote comments in the free text sections. There were 2541 visits to the study's website. Most respondents (66%) accessed the website from a referring website, 32% accessed the website through direct traffic, and 2% found the questionnaire through a search engine. The majority of the referrals came from the Facebook website. Most respondents (66%) reported hearing about the study through a website or an electronic newsletter/email.

Median age of respondents was 20 years; most were female (74%). Respondents reported residing in all Australian states and territories except the Northern Territory with most (83%) living in a major city. The majority (88%) were either currently in tertiary education or held a bachelor's degree or higher. Overall, 76% reported having penetrative (vaginal or anal) sex in the previous twelve months. The median number of reported sexual partners in that time period, for both women and men, was 1 (range: 0-28 and 0-19 respectively). A higher proportion of males reported same-sex partners (23% vs. 10%; p =< 0.01). Respondents were more likely to be female and aged 20 to 24 years than the general population of same age [21], and males were more likely to report male-to-male sexual contact than similarly aged males [22] (Table 1).

**Table 1:** Demographic, health care access, and sexual behaviour characteristics of the sample

Characteristic		N (%)	CI (95%)	**Reference Population**^**1**^
**Age**	16 to 19	214 (32%)	(29%-36%)	44%^2^

	20 to 24	448 (68%)	(64%-71%)	56%

**Gender**	Female	487 (74%)	(70%-77%)	49%^2^

	Male	173 (26%)	(23%-30%)	51%

**Aboriginal or Torres Strait Islander**	Yes	7 (1%)	(0%-2%)	2%^2^

	No	655 (99%)	(98%-100%)	98%

**Remoteness**	Major city^3^	548 (83%)	(80%-86%)	84%^4^

	Non-major city^3^	111 (17%)	(14%-20%)	16%

**Country born**	Australia	515 (78%)	(75%-81%)	78%^2^

	Other	147 (22%)	(19%-25%)	22%

**Education**	Did not complete high school	6 (1%)	(0%-2%)	---+^5^

	Still studying high school	23 (4%)	(2%-5%)	45%

	Completed high school and not studying at TAFE or tertiary degree	24 (4%)	(2%-5%)	---+

	Still studying or completed TAFE	27 (4%)	(3%-6%)	---+

	Still studying tertiary or Bachelor's degree or higher	582 (88%)	(85%-90%)	20%

**Women: any same-sex partners**	Yes	38 (8%)	(5%-10%)	10% (16-19 yrs)^6 ^12% (20-29 yrs)

	No	449 (92%)	(90%-95%)	

**Men: any same-sex partners**	Yes	29 (17%)	(11%-22%)	2% (16-19 yrs) ^6 ^7% (20-29 yrs)

	No	144 (83%)	(78%-89%)	

**Number sexual partners in prior 12 months**	Men with no same-sex partners	1.62 (mean) 1 (median) 0-19 (range)	1.22-2.02 (mean)	1.3 (mean, 16-19 yrs)^7 ^1.5 (mean, 20-29 yrs)

	Women with no same-sex partners	1.44 (mean) 1 (median) 0-12 (range)	1.28-1.60 (mean)	1.0 (mean, 16-19 yrs) 1.1 (mean, 20-29 yrs)

**Women: Past STI diagnosis**	Yes	38 (8%)	(5%-10%)	3% (aged 16-19)^8 ^12% (aged 20-29)

	No	449 (92%)	(90%-95%)	

**Men: Past STI diagnosis**	Yes	4 (2%)	(0%-5%)	1% (aged 16-19)^8 ^11% (aged 20-29)

	No	169 (98%)	(95%-100%)	

^1^Demographic data were compared to the Census data and the Australian Study of Health and Relationships data for similarly aged men and women.

^2 ^Census data [21]

^3^Remoteness defined in accordance with the Australian Standard Geographical Classification-Remoteness Area System in 2010. Major city in the study is defined as RA1; Non-major city is defined as RA2-RA5 [29].

^4^Census data [30]

^5 ^Direct comparisons to data provided when available from Census data [21,31]. ^+^Symbol denotes comparable data are not available.

^6 ^Australian Study of Health and Relationships data [22]

^7 ^Median and range not available for the Australian Study of Health and Relationships data [32].

^8^Australian Study of Health and Relationship data [33]

Thirty four percent had had a past STI test, with 19% (n = 42) of this group reporting being diagnosed with an STI. Fifteen percent (n = 102) of respondents agreed with the statement "I feel I could be at risk for a sexually transmitted infection (STI)".

#### Access to a doctor

Women were more likely to have consulted a doctor in the last 12 months, with a median of 4 visits compared with 2 for men (p < 0.01). Twenty eight percent (n = 185) reported that they found it difficult to access a doctor with whom they would be willing to discuss a sexual health concern and 85% (n = 158) of these said that the main reason was not feeling comfortable talking to the local doctor about a sexual health concern. Respondents in their 20 s (p =< 0.01), those born in Australia (p =< 0.01), and those with three or more sexual partners in the previous year reported finding it easier to access a doctor than respondents in their teens, those born outside Australia, and those with fewer than three sexual partners in the previous year.

### Willingness to have a sexual health consultation by different media

Overall, 85% of the sample indicated they would be willing to have an in-person consultation with a doctor, 63% a telephone consultation, and 29% a webcam consultation (Table 2). Some differences were found in how willing respondents were to speak to a doctor by different media (Table 3). It is notable that respondents who had had an STI test in the past were more willing to have an in-person consultation than respondents who had never had an STI test (94% vs. 84%, p = 0.01). Men were more willing than women to have a webcam consultation (36% vs. 26%, p = 0.01), as were respondents who reported same-sex partners compared with those with no same-sex partners (45% vs. 27%, p =< 0.01). Additionally, respondents reporting three or more partners were more willing to have a webcam consultation than respondents reporting fewer partners (38% vs. 27%, p = 0.01).

**Table 2:** Responses to questions regarding access to sexual health services and views on telemedicine by gender

Question		Women	Men	Total
**Difficulty accessing a doctor for a sexual health concern**	Easy	229 (47%)	78 (45%)	308 (47%)

	Neither easy nor difficult	117 (24%)	52 (30%)	169 (26%)

	Difficult	141 (29%)	43 (25%)	185 (28%)

**Access to a webcam**	Yes	333 (68%)	118 (68%)	453 (68%)

	No	154 (32%)	55 (32%)	209 (32%)

**Willingness to have an in-person sexual health consultation**	Willing	410 (84%)	151 (87%)	563 (85%)

	Unwilling	77 (16%)	22 (13%)	99 (15%)

**Willingness to have a telephone sexual health consultation**	Willing	297 (61%)	119 (69%)	417 (63%)

	Unwilling	190 (39%)	54 (31%)	245 (37%)

**Willingness to have a webcam sexual health consultation**	Willing	127 (26%)*	63 (36%)*	192 (29%)

	Unwilling	360 (74%)	110 (64%)	470 (71%)

**Top preference for type of sexual health consultation if imagining one lived 20 minutes from a clinic**	In person	407 (84%)	138 (80%)	547 (83%)

	Telephone	73 (15%)	28 (16%)	101 (15%)

	Webcam	7 (1%)	7 (4%)	14 (2%)

**Top preference for type of sexual health consultation if imagining one lived 2 hours from a clinic**	In person	188 (39%)	66 (38%)	255 (39%)

	Telephone	258 (53%)	82 (47%)	340 (51%)

	Webcam	41 (8%)	25 (15%)	67 (10%)

**Willingness to receive testing kits/treatment in post**	Willing	430 (88%)	148 (86%)	580 (88%)

	Unwilling	57 (12%)	25 (14%)	82 (12%)

**Prefers another mode to speak to a doctor**	No	240 (49%)	90 (52%)	330 (50%)

	Instant messaging	79 (16%)	39 (23%)	119 (18%)

	E-mail	158 (32%)	43 (25%)	202 (31%)

	SMS	4 (1%)	1 (1%)	5 (1%)

*Indicates a statistically significant difference-chi-square test

**Table 3:** Factors associated with willingness to have a sexual health consultation by different media

		In person			Telephone			Webcam		
**Variable**		**Willing**	**Unwilling**	p value*	**Willing**	**Unwilling**	p value*	**Willing**	**Unwilling**	p value*

**Gender**	Male	151 (87%)	22 (13%)	0.33	119 (69%)	54 (31%)	0.07	63 (36%)	110 (64%)	**0.01**

	Female	410 (84%)	77 (16%)		297 (61%)	190 (39%)		127 (26%)	360 (74%)	

**Age**	16 to 19	173 (81%)	41 (19%)	**0.04**	147 (69%)	67 (31%)	**0.04**	63 (29%)	151 (71%)	0.86

	20 to 24	390 (87%)	58 (13%)		270 (60%)	178 (40%)		129 (29%)	319 (71%)	

**Remoteness**	Major city	474 (87%)	74 (14%)	**0.03**	353 (64%)	195 (36%)	0.06	158 (29%)	390 (71%)	1

	Non-major city	87 (78%)	24 (22%)		61 (55%)	50 (45%)		32 (29%)	79(71%)	

**Had an STI test**	Yes	207 (93%)	16 (7%)	**<0.01**	140 (63%)	83 (37%)	0.9	66 (30%)	157 (70%)	0.8

	No	351 (81%)	82 (19%)		274 (63%)	159 (37%)		124 (29%)	309 (71%)	

**Any same-sex partners**	No	498 (84%)	95 (16%)	**0.03**	365 (62%)	228 (38%)	**0.02**	160 (27%)	433 (73%)	**<0.01**

	Yes	63 (94%)	4 (6%)		51 (76%)	16 (24%)		30 (45%)	37 (55%)	

**Yearly visits to a doctor**	0 to 3	303 (85%)	55 (15%)	0.75	234 (65%)	124 (35%)	0.17	111 (31%)	247 (69%)	0.22

	4+	260 (86%)	44 (15%)		183 (60%)	121 (40%)		81 (27%)	223 (73%)	

**Partner total**	0-2	440 (84%)	86 (16%)	0.05	327 (62%)	199 (38%)	0.39	141 (27%)	385 (73%)	**0.01**

	3+	123 (90%)	13 (10%)		90 (66%)	46 (34%)		51 (38%)	85 (63%)	

*Chi-square test

Sixty eight percent (n = 453) of the sample reported having access to a webcam they could use for a sexual health consultation. Of those who did not own a webcam, only 13% (n = 26) reported being willing to purchase a webcam for this purpose. There was no association between owning a webcam and willingness to have a webcam consultation (p = 0.30).

#### Free text responses

In addition to the forced-choice answers to the questions in this section, respondents were invited to comment in the free text boxes. The free text responses provided further insight into young people's views on telemedicine consultations. Three main advantages of telephone consultations were identified: 1) patients could remain anonymous; 2) the telephone was deemed a more convenient and less embarrassing medium for speaking to a doctor than in person; and 3) such consultations were assessed as saving time because no travel to a clinic was required (Table 4). Concerns about telephone consultations included difficulty verifying the doctor's credentials and the potential for eavesdropping.

**Table 4:** Free text examples of perceived advantages and disadvantage of telemedicine consultations

**Advantages of telephone consultations**
*i. Patients can remain anonymous*
"By communicating over the phone i'd probably be more willing to discuss private details and be able to feel somewhat anonymous." (Female, aged 23)
*ii. Telephone consultations are less embarrassing and more convenient than in-person consultations*
"Over the phone is far less embarrassing." (Female, aged 20)
"Telephone consults would help a lot, especially if there was a short waiting time. I hate GP waiting rooms." (Male, aged 21)
*iii. Time saving*
"The idea of communicating from home would in many cases be easier- no travel, less time wasted." (Female, aged 24)

**Disadvantages of telephone consultations**
*i. Difficulty verifying the doctor's credentials and the potential for eavesdropping*
"Over the phone is probably a less appealing option because you dont [sic] know who exactly you are talking to, or if others are listening in." (Male, aged 19)

**Advantages of webcam consultations**
*i. Enables face-to-face engagement with the doctor*
I would be much more comfortable with a webcam than over the phone as there's much more of a sense of face-to-face contact. (Female, aged 20)
"Great idea. Confort [sic] of your own home, but you would be able to see that the doctor is in their office in a confidential environment." (Female, aged 21)
ii. No need to travel to a clinic
"I think [a webcam consultation is] a great idea, it would save people having to make the trip to the medical centre." (Female, aged 18)

**Disadvantages of webcam consultations**
*i. Privacy and security concerns*
"The reason I would feel uncomfortable about using a webcam would be that I would fear someone could hack into my computer and access the chat between my GP and I. Obviously for confidentiality reasons this would be disasterous [sic]." (Female, aged 24)
"I would be concerned about the retention of webcam data. The Doctor would need to have a policy about this. Preferable [sic] the policy would be never keep [sic] any permanent record of any data ever. If enough of this data exists it is inevitable that some of it will be misplaced or stolen at some point." (Male, aged 23)
*ii. Viewing webcam consultations as unnecessary*
"I don't see the point of using a webcam - if it's something that can be discussed at a distance, then the telephone should suffice. If it's something that needs to be done with visual interaction, surely it should be done in person." (Female, aged 23)

The main objections to webcam consultations were privacy and security concerns about the possibility of the webcam consultation being recorded, saved, and potentially searchable and retrievable online (Table 4). Others found webcam consultations unnecessary because telephone was adequate if the consultation could occur at a distance. Despite these objections, a few respondents reported viewing webcam consultations as advantageous either because they avoided travel or because, unlike the telephone, webcam consultations enabled face-to-face engagement with the doctor.

### Preferred medium for an asymptomatic consultation

If imagining they lived 20 minutes from a doctor, 83% of respondents reported their first preference as an in-person consultation with a doctor, 15% preferred telephone, and only 2% webcam (Table 2). Multivariate analysis revealed that respondents who had never had an STI test had an increased odds of choosing speaking to a doctor by telephone as their top preference when compared to an in-person consultation (OR 1.84; 95% CI 1.08-3.14) (Table 5). Additionally, respondents with three or more partners had increased odds of preferring to speak to a doctor over webcam compared with an in-person consultation (OR 4.24; 95% CI 1.24-14.42). No other associations were found.

**Table 5:** Repondents' preferred medium for consulting a doctor if hypothetically living 20 minutes from a clinic

			20 minutes							
			**Telephone**^**1**^				**Webcam**^**1**^			

**Variable**		**n%**	**Unadjusted OR (95% CI)**	**p value**^**2**^	**Adjusted OR**^**3 **^**(95% CI)**	**p value **^**2**^	**Unadjusted OR (95% CI)**	**p value**^**2**^	**Adjusted OR**^**3 **^**(95% CI)**	**p value**^**2**^

**Sex**	Female^4^	487 (74%)	1		1		1		1	

	Male	173 (26%)	1.13 (0.70-1.82)	0.61	1.08 (0.65-1.79)	0.78	2.95 (1.02-8.56)	0.05	2.26 (0.68-7.57)	0.19

**Age**	16 to 19	214 (32%)	1.10 (0.70-1.72)	0.69	0.97 (0.61-1.54)	0.89	2.16 (0.75-6.26)	0.16	3.22 (0.97-10.75)	0.06

	20 to 24^4^	448 (68%)	1		1		1		1	

**Remoteness**	Major city^4^	548 (83%)	1		1		1		1	

	Non-major city	111 (17%)	0.84 (0.46-1.51)	0.55	0.83 (0.45-1.50)	0.53	0.80 (0.18-3.62)	0.77	0.77 (0.16-3.68)	0.74

**Had an STI test**	Yes^4^	223 (34%)	1		1		1		1	

	No	433 (65%)	1.91 (1.16-3.13)	**0.01**	1.84 (1.08-3.14)	**0.03**	0.90 (0.29-2.79)	0.85	0.75 (0.19-2.92)	0.68

**Any same-sex partners**	Yes	67 (10%)	0.75 (0.35-1.63)	0.47	0.94 (0.42-2.11)	0.88	2.38 (0.65-8.80)	0.19	1.13 (0.25-5.07)	0.87

	No^4^	593 (90%)	1		1		1		1	

**Yearly visits to a doctor**	0-3^4^	358 (54%)	1		1		1		1	

	4+	304 (46%)	0.97 (0.64-1.49)	0.90	1.10 (0.70-1.73)	0.67	0.65 (0.21-1.95)	0.44	0.86 (0.26-2.91)	0.81

**Partner total**	0-2^4^	526 (80%)	1		1		1		1	

	3+	136 (21%)	0.66 (0.37-1.19)	0.66	0.78 (0.42-1.44)	0.42	3.80 (1.31-11.05)	**0.01**	4.24 (1.24-14.42)	**0.02**

										

^1^Reference category is in-person consultation

^2^Chi-square test

^3^Multinominal regression. Adjusted for sex, age, remoteness, past STI test, same-sex partners, doctor visits, and partner total.

^4^Reference category

When respondents were asked to imagine that they lived two hours from a doctor, most (51%) preferred a telephone consultation (Table 2). Thirty nine percent indicated that an in-person consultation was their top preference and 10% preferred webcam. No associations were found (Table 6).

**Table 6:** Repondents' preferred medium for consulting a doctor if hypothetically living 2 hours from a clinic

			2 hours							
			**Telephone**^1^				**Webcam**^1^			

**Variable**		**n%**	**Unadjusted OR (95% CI)**	**p value**^**2**^	**Adjusted OR**^**3 **^**(95% CI)**	**p value**^**2**^	**Unadjusted OR (95% CI)**	**p value**^**2**^	**Adjusted OR**^**3 **^**(95% CI)**	**p value**^**2**^

**Sex**	Female^4^	487 (74%)	1		1		1		1	

	Male	173 (26%)	0.91 (0.62-1.32)	0.60	0.85 (0.57-1.27)	0.43	1.74 (0.98-3.07)	0.06	1.63 (0.87-3.08)	0.13

**Age**	16 to 19	214 (32%)	0.92 (0.65-1.30)	0.63	0.90 (0.63-1.29)	0.56	0.98 (0.55-1.73)	0.94	1.02 (0.55-1.90)	0.94

	20 to 24^4^	448 (68%)	1		1		1		1	

**Remoteness**	Major city	548 (83%)	1		1		1		1	

	Non-major city	111 (17%)	0.97 (0.63-1.49)	0.87	1.02 (0.66-1.60)	0.92	0.85 (0.40-1.80)	0.68	0.98 (0.46-2.11)	0.97

**Had an STI test**	Yes^4^	223 (34%)	1		1		1		1	

	No	433 (65%)	0.95 (0.67-1.34)	0.77	0.92 (0.63-1.34)	0.92	0.73 (0.42-1.28)	0.27	0.64 (0.34-1.20)	0.16

**Any same-sex partners**	Yes	67 (10%)	0.87 (0.51-1.50)	0.62	0.85 (0.48-1.50)	0.85	1.16 (0.50-2.69)	0.73	0.84 (0.33-2.16)	0.73

	No^4^	593 (90%)	1		1		1		1	

**Yearly visits to a doctor**	0-3^4^	358 (54%)	1		1		1		1	

	4+	304 (46%)	0.77 (0.55-1.06)	0.77	0.72 (0.51-1.02)	0.06	0.82 (0.48-1.40)	0.46	0.88 (0.49-1.58)	0.67

**Partner total**	0-2^4^	526 (80%)	1		1		1		1	

	3+	136 (21%)	0.99 (0.66-1.49)	0.98	0.99 (0.64-1.52)	0.95	1.13 (0.59-2.16)	0.72	0.98 (0.48-1.99)	0.95

^1^Reference category is in-person consultation

^2^Chi-square test

^3^Multinominal regression. Adjusted for sex, age, remoteness, past STI test, same-sex partners, doctor visits, and partner total.

^4^Reference category

### Other modes of communication with a doctor

Respondents were asked if there was another mode of communication they would prefer to use to speak to a doctor about a sexual health matter. Fifty percent (n = 330) said no, 31% (n = 202) said they preferred email, 18% preferred instant messaging, and 1% preferred SMS.

### Home STI testing kits

Eighty eight percent (n = 580) of the sample was willing to receive testing kits and/or treatment through the post.

## Discussion

This study is the first we are aware of to seek the views of young people on telemedicine and access to sexual health services for STI care. The survey revealed that most young people would not use webcam consultations, because they had strong concerns about the inherent confidentiality and security. However, a minority did express a more favourable view. Men, respondents with same-sex partners, and respondents with three or more sexual partners reported finding webcam consultations more acceptable. Respondents overall were more favourably disposed to telephone consultation and most were willing to receive home STI tests and treatment through the post.

These results highlight the value of offering a variety of options for accessing sexual health services in order to cater to heterogeneous needs. While only about a third of respondents were willing to consult by webcam, such a service may be invaluable for youth who may not otherwise access a sexual health service. More research is needed to improve understanding of the circumstances in which particular subsets of young people find webcam consultations most acceptable.

The acceptability of webcam consultations may be increased by mitigating perceived privacy and security concerns. For example, clinics could clearly state their policy that webcam consultations would never be recorded or saved. Consultations could be conducted over an encrypted Internet connection for increased security. A comprehensive and comprehensible security and privacy policy would ideally be easily located on the medical centre's website as well as reiterated before every online consultation [23]. Additionally, an attractive, professionally designed website may increase people's trust in the medical centre's service [23]. Such tactics have been successfully used to increase the acceptability of and trust in other types of sensitive online transactions, such as online banking [23], and may similarly increase the acceptability of online medical consultations.

It is possible that security concerns could be lessened if the consultation were not directly between the doctor and the patient in their home, but rather, as in other telemedicine services, between a distant specialist and a health care professional together with a patient in a local clinic. In this situation the service may be perceived as more legitimate and the doctor on the screen as more trustworthy because the consultation is validated by taking place in a clinic.

The privacy and security concerns expressed about webcam consultations are not specific to sexual health. The larger telemedicine literature reveals that patients commonly express privacy and security concerns about using this technology to consult a doctor [8,12]. It has also been argued that patients may be more apprehensive about their privacy during a webcam consultation compared with an in-person consultation, because there are no standards currently in place to guarantee patients' privacy and security when their health information is transmitted online [8]. A qualitative study examining people's pre-use views of webcam consultations for general health matters also reported that participants were concerned that, once the consultation was transmitted online, security measures could be breached and the footage could become accessible to anybody [24].

Results from other telemedicine studies suggest that webcam consultations for sexual health may be most successful in two scenarios. The first is using webcam consultations for follow-up appointments, in which the client would likely already have a trusting relationship with the health care professional. A qualitative study examining HIV/AIDS patients' use of home telemedicine, for example, reported that, although patients were willing to have webcam consultations, they preferred first consultations to be in person in order to develop a relationship with the health care professional, which was perceived as difficult to do over a webcam [25]. Using webcam consultation in a similar manner for STI care may increase its acceptability.

The second situation where there may be value in a webcam consultation is psychological counselling following the diagnosis of an STI; mental health is one field where telemedicine has frequently been applied [10]. Reviews have found that mental health services provided to patients over video are highly reliable in comparison with in-person consultations, and that patients report high levels of satisfaction with these services [10]. Webcam consultations could thus be used when informing patients of a positive diagnosis.

The current study has some limitations. First, the results are from a self-selected convenience sample, not a representative sample. Some recruitment strategies were more successful than others. However, we have no evidence to explain why some organisations were more willing than others to advertise. It is possible that some organisations were deterred by the sensitive topic of youth's sexual health; STI services have been perceived as "unmentionable" or controversial topics in advertising [26,27]. Comparison to the Census data reveals that women were overrepresented. Given that women in the study reported being less willing than men to have a webcam consultation, the general population may find webcams slightly more acceptable. Most respondents also had high levels of education. In Australia, people with high levels of education have higher rates of home Internet access [28]. Greater access to and familiarity with the Internet could result in a sample more able and willing to have a webcam consultation than the general population. However, such a sample may also be more aware than the general population of the security and confidentiality risks posed by an online service. The second limitation is that the study asked people's hypothetical views on using a telemedicine service. People's views on such service may vary if they were actually using the service. However, pre-use views are important in helping to determine whether such services should be implemented. Finally, the results from the study cannot be generalized beyond the field of sexual health.

## Conclusion

While the majority of respondents were willing to have a telephone consultation, only 29% were willing to have a webcam consultation for sexual health. Although the acceptability of webcam consultations is currently low, efforts to reduce privacy and security concerns may help to augment the acceptability of such services and will influence whether webcam consultations are eventually adopted on a large scale. Furthermore, the value of webcam services to an important minority of youth should not be overlooked.

## Competing interests

The authors declare that they have no competing interests.

## Authors' contributions

CCG designed the study, advertised, collected the data, conducted the data analysis and drafted the manuscript under the guidance of her supervisors. Supervisors JH, MYC, CKF, and MK (principal supervisor) contributed to the design of the study, assisted in the interpretation of the data, and revised the manuscript. All authors read and approved the final manuscript.

## Pre-publication history

The pre-publication history for this paper can be accessed here:

http://www.biomedcentral.com/1471-2334/11/285/prepub
